# Bilateral Severely Stenotic Jugular Foramen: Diagnosis and Management from the Otologist/Neurotologist Point of View

**DOI:** 10.1155/2020/1530310

**Published:** 2020-06-04

**Authors:** Raquel Acinho Bento, David Rodrigues, João Levy, Tiago Eça, Vitor Oliveira, Leonel Luís

**Affiliations:** ^1^Department of Otorhinolaryngology, Centro Hospitalar Universitário Lisboa Norte, Lisbon, Portugal; ^2^Department of Neuroradiology, Centro Hospitalar Universitário Lisboa Norte, Lisbon, Portugal

## Abstract

Bilateral jugular foramen stenosis with jugular bulb and vein aplasia is rare in nonsyndromic craniosynostosis and usually diagnosed during childhood. We present a case of bilateral jugular foramen stenosis with jugular bulb and vein aplasia, with subsequent persistence and enlargement of the fetal venous anastomosis in the middle and posterior cranial fossa, along with a review of the literature about this anatomical abnormality, highlighting the surgical challenges and management from the otologist/neurotologist point of view.

## 1. Introduction

Internal jugular vein (IJV) drains blood from the head and neck region and is a major contributor to venous drainage from intracranial structures [1].

Emissary veins (EV) are valveless veins that pass through cranial openings, complementing the IJV in the extracranial venous drainage of the posterior fossa dural sinuses [2]. They represent the remaining connections of the superficial venous system with the deep dural system during the development of the skull [3].

The prevalence of the major posterior fossa EV is not low in the general population [2], being usually small and asymptomatic. Although EV can protect the brain from rises of intracranial temperature (helping to cool venous blood circulating through cephalic structures) and pressure (in patients with lesions of the neck or skull base and obstructed IJV) [4], they play a minor role in the people whose jugular venous system is intact [5].

EV may be enlarged in the event of high-flow vascular malformations or in severe IJV aplasia or thrombosis, as they became the only pathway for draining the blood from the posterior fossa, converting the external jugular vein to the foremost encephalic outflow pathway [6].

EV may be of clinical importance to otologists not only because they may present with pulsatile tinnitus but also because they may cause a significant intraoperative bleeding or postoperative sinus thrombosis [2, 7]. Furthermore, their presence can allow retrograde spread from external ear canal infection or tumour [6].

We present a case report of bilateral jugular foramen stenosis with jugular bulb and vein aplasia and review the literature about this anatomical abnormality, highlighting the surgical challenges and management from the otologist/neurotologist point of view.

## 2. Case Presentation

A 40-year-old woman presented in our hospital with a 10-year bilateral progressive hearing loss. She had been diagnosed with bilateral otosclerosis at another hospital.

The patient had an unspecified head surgery during childhood but no other relevant medical history, namely no history of noise trauma, ototoxic drugs, or relevant familiar history.

She had a normal otologic and head and neck examination with no syndromic features.

Audiology testing revealed a mild bilateral mixed hearing loss (15 dB mean air-bone gap), an ipsilateral type as tympanogram bilaterally and absent ipsilateral acoustic reflexes.

**Figure 1:**
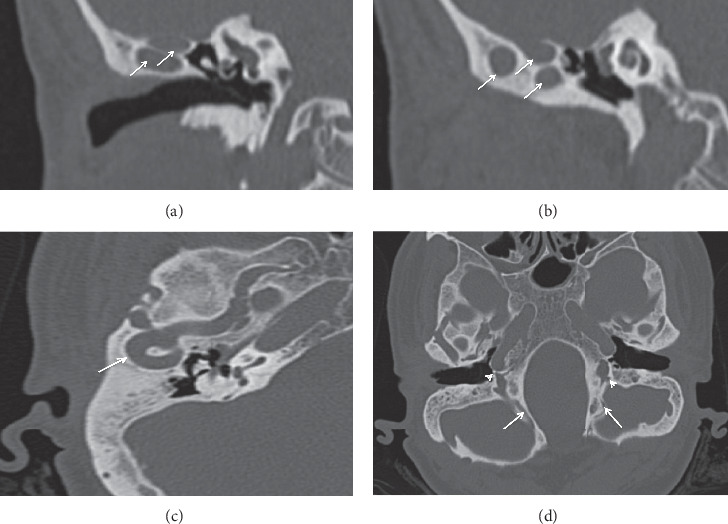
Imaging of the right ear showing enlarged emissary veins (arrows). (a), (b) Coronal HRCT. (c) Axial HRCT. (d) Axial HRCT (mIP), showing small posterior condylar emissary canal on the left (jugular bulb type) and groove on the right (occipital sinus type)—arrows; atresic jugular foramen—arrow heads. HRCT: high-resolution computed tomography; mIP: maximum intensity projection.

**Figure 2:**
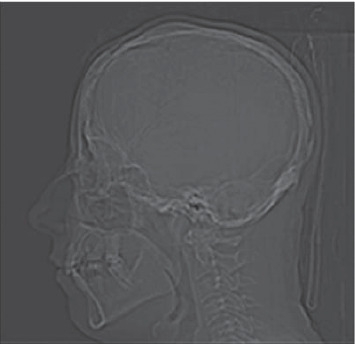
Lateral scanogram: irregular thickness in the calvarium contour suggestive of a previous cranioplasty in childhood due to craniossinostosis.

A preoperative high-resolution computed tomography (HRCT) scan was suggestive of bilateral superior semicircular canal dehiscence and showed a bilateral jugular bulb and foramen aplasia, a persistent petrosquamous sinus, and multiple vascular impressions in the middle cranial fossa, the latter causing a tortuous tubular images projecting in the proximity of the temporomandibular joints and separated from the superior and posterior wall of the external ear canal by only a thin bony lamella (Figures 1(a)–1(c). Irregular thickness in the calvarium contour suggestive of a previous cranial surgery was also identified on CT scans (Figure 2).

**Figure 3:**
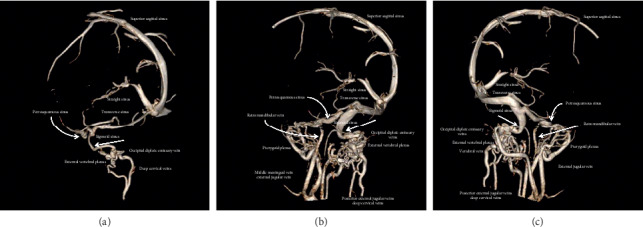
MRV; (a), (b) right circulation, (c) left circulation. MRV: magnetic resonance venography.

**Figure 4:**
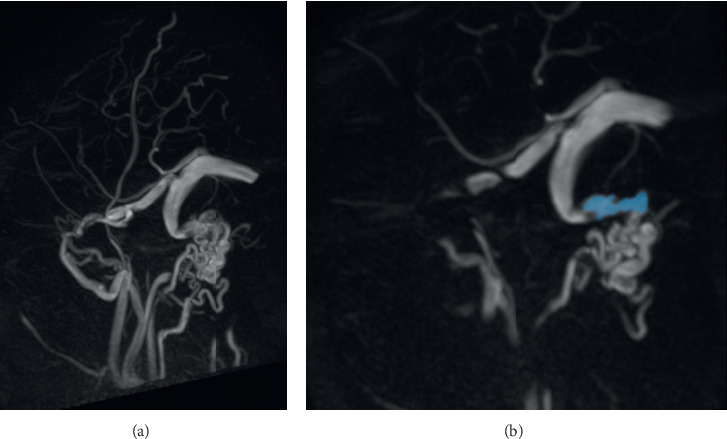
Sagital MRV broad (a) and detailed (b) mIP, depicting right emissary mastoid vein (blue) draining right sigmoid sinus into posterior auricular vein and suboccipital plexus.

**Figure 5:**
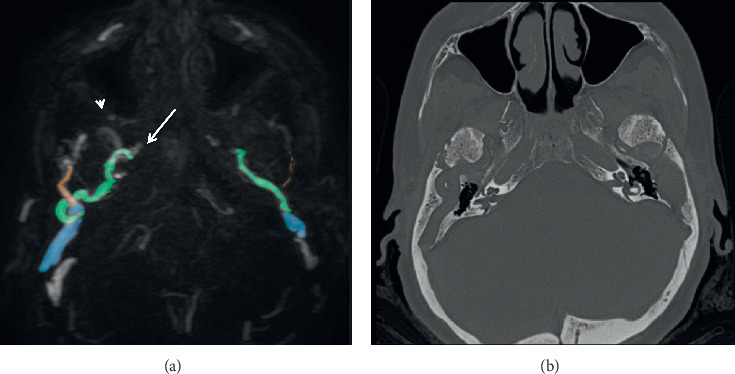
Axial MRV (a) and HRCT mIP (b) showing striking asymmetry in the petrosquamosal sinuses (PSS) and canals, bilaterally (blue); middle meningeal sinus (green) draining into the cavernous sinus (arrow) and pterygoid venous plexus (arrow head) via the foramen ovale; retromandibular vein (orange) draining into external jugular vein; mastoid emissary vein (white) posterior to PSS.

Further imaging with magnetic resonance venography (MRV) showed a right dominant transverse and sigmoid venous sinus, a bilateral internal jugular bulb, and vein aplasia. It further identified a bilateral persistent petrosquamous sinus (draining into a retromandibular vein, via retroauricular foramen and pterygoid plexus, and via foramen oval), a posterior condylar emissary vein (draining into the external vertebral venous plexus and via condylar foramen), a mastoid emissary vein (draining into the posterior auricular vein and mastoid foramen) and an occipital emissary vein (draining into the suboccipital plexus) (Figures 3(a)–3(c); Figures 4(a) and 4(b); and Figures 5(a) and 5(b)).

Both cervical and ocular vestibular evoked myogenic potentials were normal.

The patient refused any otological treatment. Furthermore, since there were not any other neurologic symptoms or signs, no further management was needed.

## 3. Discussions

Jugular and sigmoid sinus are relatively narrow, reaching adult size only two years after birth, probably as a result of haemodynamic factors caused by the adoption of an upright posture. Until then, EV play a role complementing the IJV system with most disappearing as sigmoid sinus and jugular vein grow, as discussed by Jeevan et al. [8].

However, EV can persist and be abnormally enlarged due to failure of normal maturation of the sigmoid-jugular complex (SJC). The cause for this anatomical abnormality is still unknown. According to Jeevan et al. [8], while one theory hypothesizes that an abnormal development of the osseous and cartilaginous structures of the jugular foramen is the main cause, another advocate a primary vascular abnormality for SJC narrow lumen. Both theories find support in craniosynostosis patients. The first theory is supported by the presence of a smaller jugular foramen in syndromic and complex craniosynostosis patients compared to healthy control patients [8–10]. The second theory is based on expression of fibroblast growth factor receptor gene products which has been identified in cranial vascular endothelia of patients with syndromic craniosynostosis as well as in cranial tissues of the developing craniosynostotic sutures [8].

There are few case series reporting EV in patients with craniofacial syndromes, with Jeevan et al. [8] reporting one study of eleven syndromic craniosynostosis patients, showing only three cases of transosseous venous drainage due to complete bilateral jugular foramen stenosis. Moreover, there are even fewer reports of complete bilateral jugular foramen stenosis in nonsyndromic craniosynostosis [11, 12]. Despite jugular foramen stenosis being found more commonly in syndromic and complex craniosynostosis cases, bilateral complete stenosis is rare even in these patients [8].

In the present case, the vascular abnormality was incidentally found during a preoperative temporal bone CT imaging in an adult patient. We presume that the patient is a nonsyndromic craniossynostosis, as there is an irregular thickness in the calvarium contour suggestive of a previous cranioplasty eventually due to a craniosynostosis, but no syndromic features (Figure 2).

CT scans revealed a jugular foramen stenosis and prompted further study of venous anatomy with MRV and 3D reconstructions. The latter showed a jugular bulb and vein aplasia and subsequent persistence and enlargement of the fetal venous anastomosis in the middle and posterior cranial fossa. These reconstructions are helpful for visualising foramen patency and specific routes of emissary drainage, although CT venography can also be reliable in depicting the intracranial venous circulation with high quality, with the disadvantage of radiation exposure [2, 12].

Mastoid emissary vein, posterior condylar vein, petrosquamosal sinus, and occipital emissary vein are the major posterior fossa emissary veins. Mastoid emissary vein originates from the sigmoid sinus and drains into the posterior auricular or occipital vein. Posterior condylar vein arises from the jugular bulb and drains into the deep cervical vein connecting also with the vertebral artery venous plexus. Occipital emissary vein arises from the transverse sinus and drains to the occipital vein and internal vertebral plexus. Petrosquamosal sinus arises from the transverse or sigmoid sinus and drains either into the retromandibular vein or into the pterygoid venous plexus [2, 8]. Detection of these vessels is crucial, since they may constitute a surgical challenge, not only due to the close relationship with the mastoid and external ear canal but also because their disruption can lead to a significant haemorrhage or a fatal rise in the intracranial pressure. For the latter reason, according to some authors [8, 12], detection of abnormally enlarged emissaries serving as major sources of intracranial venous drainage may contraindicate surgical intervention. In conclusion, preoperative imaging delivers a detailed study of anomalous transosseous venous drainage, not only for the evaluation of morbidity and mortality risks but also for the preoperative planning, allowing modification of surgical approaches to reduce these associated risks.

Our patient had hypoacusia and no other morbidities related to this anatomical variation, so no further management was offered. The patient further refused any surgical approach or hearing rehabilitation.

In cases where surgical management is warrant (mastoid or lateral skull base surgery), EV may present as a surgical challenge, as they may lead to a dramatic bleeding due to their large diameter [13, 14]. According to An et al. [13], several methods for hemostasis can be used: drilling with a diamond burr, packing with hemostatic material, electrocautery, vessel ligation, and bone wax application.

If in small and medium diameter EV, using electrocautery or plugging EV foramen with absorbable hemostatic material or bone wax seem an appropriate method of hemostasis, in large EV it may be necessary a free graft of connective tissue (e.g., muscle) plug, suture ligation of the vein (after being skeletonized through drilling), or a pedicled flap (indented into the EV foramen) [14].

The different approach to different EV sizes may be justified by the increased risk of bone wax migrating into the lateral sinus, especially in abnormally dilated, short, and straight EV. Additionally, unipolar electrocautery can be contraindicated in the presence of implanted electronic devices, namely cochlear implants [13, 15].

In conclusion, we present a case of a bilateral jugular foramen stenosis associated with a persistence and enlargement of the fetal venous anastomosis in the middle and posterior cranial fossa. This was incidentally diagnosed with imaging studies in an asymptomatic adult patient with a presumed nonsyndromic craniosynostosis. The thorough knowledge of these anatomical abnormalities is essential for a correct diagnosis as well as for the otological surgical preoperative planning and management of intraoperative complications.
